# Back to the Future: Reintroduction into the Wild of the Italian Grey Partridge (*Perdix perdix italica* Hartert, 1917)

**DOI:** 10.3390/ani16111685

**Published:** 2026-05-30

**Authors:** Francesco Riga, Chiara Gabbrielli, Claudia Greco, Paolo Montanaro, Nadia Mucci, Davide Senserini, Cristiano Tabarroni, Daniel Tramontana, Stefania Volani

**Affiliations:** 1 Unit for Technical Advice and Strategies for Conservation and Management of National Wildlife and Mitigation of Damages and Impacts (BIO-CFN), Italian Institute for Environmental Protection and Research (ISPRA), 00144 Rome, Italy; paolo.montanaro@isprambiente.it (P.M.); stefania.volani@gmail.com (S.V.); 2 Italian Hunting Federation (FIdC), 00198 Roma, Italy; chia.gabbrielli@gmail.com (C.G.); davidesenserini@gmail.com (D.S.); daniel.tramontana@fidc.it (D.T.); 3 Research Unit of Beahavioural Ecology, Ethology and Wild Life Management, Department of Life Sciences, University of Siena, 53100 Siena, Italy; 4 Unit for Conservation Genetics (BIO-CGE), Italian Institute for Environmental Protection and Research (ISPRA), 40064 Ozzano dell’Emilia, Italy; claudia.greco@isprambiente.it (C.G.); nadia.mucci@isprambiente.it (N.M.); cristiano.tabarroni@isprambiente.it (C.T.); 5 Department of Civil, Environmental and Mechanical Engineering, University of Trento, 38123 Trento, Italy

**Keywords:** Italian grey partridge, reintroduction, playback method, home range, dispersal, birds conservation

## Abstract

The Italian grey partridge (*Perdix perdix italica* Hartert, 1917) is a subspecies endemic to the Italian peninsula, considered extinct in the wild, and it is included in Annex I of the EU Bird Directive. The aim of our project is to reintroduce a viable population of the endemic taxon into a protected area in north-east Italy, using genetically selected founder birds belonging to the original Italian genetic lineage. From 2021 to 2023, a total of 30,078 partridges were reintroduced to Valle del Mezzano using a soft-release method, and 241 of them were equipped with VHF transmitters to evaluate survival, dispersal and spacing behaviour of the released birds. Furthermore, male spring counts using the playback method were performed to assess the number of reproductive pairs. The overall survival was 21.86%; the Cox proportional hazards model, including time-dependent effects, showed that neither sex (HR = 1.20, *p* = 0.28) nor body mass significantly influenced survival. The release year was included as a stratification factor, and site-level heterogeneity was negligible. These results show that survival dynamics were not explained solely by individual traits. Dispersal distance and spatial behaviour were affected by the release year, while sex had only a minor effect and body mass had no impact. Local heterogeneity contributed to variability but did not override the strong temporal pattern. The number of calling males in spring ranged from 152 to 267, with variations between years. Overall, our findings suggest that post-release performance in reintroduced individuals is influenced more by environmental factors than by inherent individual traits. In conclusion, despite high mortality rates among reintroduced birds, the first breeding nucleus of Italian partridges since their extinction in the wild has been observed.

## Simple Summary

The Italian grey partridge (*Perdix perdix italica* Hartert, 1917) is a subspecies endemic to the Italian peninsula, considered extinct in the wild, and is included in Annex I of the EU Bird Directive. The decline of the grey partridge in Italy, as in many other European populations, is driven by several changes in farming practices, including intensive pesticide use and the simplification of the agricultural landscape. Furthermore, natural habitat fragmentation, overhunting, and the large-scale release of birds belonging to non-native subspecies play important roles. With the genetic selection of farmed birds from the Italian genetic lineage, we have planned the reintroduction of the Italian grey partridge into a protected agricultural area in north-east Italy (Valle del Mezzano), characterised by a very low human population density. The reintroduction was performed using a soft-release method, with 18 release pens built and with crops suitable for the species’ feeding and recovery sown. Survival and dispersal of released birds were investigated by radiotelemetry, and the number of breeding pairs in spring was assessed with the playback method. Our results highlighted a high mortality rate among released birds, mainly due to raptor predation; however, spring pair counts confirmed the occurrence of Italian grey partridge breeding pairs.

## 1. Introduction

The grey partridge *Perdix perdix* is a non-migratory, ground-dwelling Phasianid inhabiting grassland and farmland [1] and considered to be an umbrella indicator species for agroecosystems [2]. It occurs across much of Europe and was introduced into North America [3].

The grey partridge is a polytypic species, with seven subspecies recognised in the original range [1]. However, a long history of bird introductions for hunting purposes has obscured morphological differences. In Italy, it was described as the Italian grey partridge *Perdix perdix italica* Hartert, 1917 [1]; this subspecies is endemic to Italy and included in Annex I of the EU Directive 2009/409/CEE (Birds Directive). Greco and colleagues [4] identified historical haplotypes exclusive to the Italian peninsula, supporting the existence of a distinct genetic lineage and the peninsula’s role as a glacial refugium.

Once widely distributed across continental Europe and the British Isles, it has experienced a severe decline since 1980, with a loss of over 96% of the estimated population at the start of the 20th century [2]. It is not currently considered threatened globally or in Europe by the IUCN, as it remains widespread, with an estimated European breeding population of 1,140,000 to 1,880,000 pairs [5].

The main causes of grey partridge decline are believed to include several changes in farming practices, including intensive pesticide use and the simplification of the agricultural landscape [6]. Furthermore, habitat fragmentation plays an important role. Indeed, the loss of natural soil and urbanisation are reputed to be responsible for changes in landscape connectivity for some wildlife species, and consequently for alterations in the genetic structure of animal populations [7].

In Italy, habitat degradation for the grey Partridge is driven by two geographically structured factors: reforestation in hilly and low-mountain areas and natural habitat loss in lowland areas. From 1885 to 2015, the forested area increased from almost 4,215,000 to almost 11,778,000 ha. Considering only the three official national inventories (1985, 2005, and 2015), the forest surface increased at a rate of 103,000 ha per year [8].

On the other hand, farmland in Italy declined from about 21,000,000 ha to 13,000,000 ha between 1886 and 2015; these landscape modifications significantly affected the species’ complete altitudinal range. In fact, increasing tree cover could limit the occurrence of game species [7]. Data on soil consumption [9] also highlight agricultural land degradation in lowland Italy.

Finally, overhunting [10] and large-scale release of allochthonous individuals since the beginning of the 20th century [2] have contributed to the decline of the grey partridge in Italy. As a result, the Italian endemic subspecies is extinct in the wild, and viable populations of *Perdix perdix perdix* occur only in the Gran Sasso Monti della Laga National Park [11] and the Monti Sibillini National Park [12], although even these two viable populations originated from the introduction of non-autochthonous birds. Elsewhere in Italy, the presence of grey Partridge is due to hunting releases, which do not form self-sustaining populations.

Management and conservation measures adopted at the international level are primarily based on habitat management and reintroduction projects. In particular, the Interreg North Sea Region Partridge (involving England, Scotland, Belgium, the Netherlands and Germany) is dedicated to improving best practice in agri-environment schemes to increase habitat suitability for grey partridge and other farmland birds [13].

On the other hand, reintroduction projects, focused on restoring local extinct taxa, could be an important conservation tool, not only for mammals and birds [14]. However, the success of reintroduction projects depends on the fitness of the founder subjects and their capacity to select suitable habitat.

In the case of the Italian grey partridge, reintroduction is not an optional choice, since the taxon is extinct in the wild. Indeed, the aim of our project was to restore a self-sustaining Italian grey partridge population in the wild by reintroducing genetically selected breeders to a suitable area in North-eastern Italy and monitoring the population after release to assess its dynamics.

## 2. Materials and Methods

### 2.1. Ethic

The translocation of Italian grey partridges from the ex situ conservation centre to Valle del Mezzano was carried out with the permission of the local health authorities and in accordance with the rules of the Istituto Sperimentale delle Venezie (IZSVe) to prevent the spread of avian influenza.

The reintroduction and monitoring of released birds (including the fitting of radio-transmitters) were carried out under Italian law for the conservation and hunting exploitation of homeotherm species (Law 157/92, article n. 7), which allows the National Institute for Environmental Protection and Research (ISPRA) to conduct research and manipulation of birds and mammals independently, without further authorisations.

### 2.2. Study Area and Founder Populations

**Figure 1:**
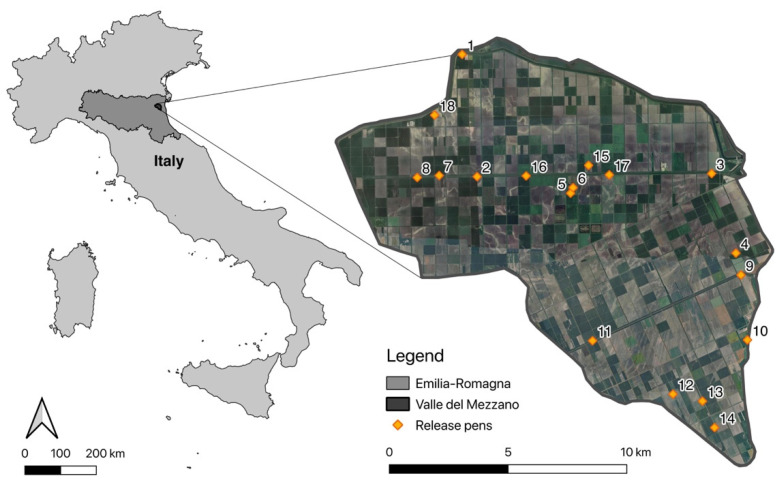
Special Protection Area Valle del Mezzano and the release pens (numbered from 1 to 18).

The study area is the Special Protection Area (SPA) IT 4060008 Valle del Mezzano (Lat 11.999236, Long 44.68435), a non-hunting area in the Emilia–Romagna Region (Figure 1). The Valle del Mezzano constituted the north-western part of the Comacchio lagoon (Po Delta), which originally covered an area of approximately 30,000 hectares. The climate in Mezzano is temperate-continental, with hot summers and very cold winters; annual temperatures range between 0 and 31 °C. Rain falls year-round in Mezzano: the month with the most rainfall in Mezzano is October, with an average rainfall of 61 mm; the month with the least rainfall in Mezzano is January, with an average rainfall of 28 mm. The altitude range between 0 and 3 m a.s.l.

As a result of extensive land reclamation, the land is divided into large-scale cultivation areas and colonised by individual rural settlements without residential structures (about 17,000 ha), formally divided into two zones: ‘South-East’ (9700 hectares) and ‘North-West’ (7200 hectares). The site is not urbanised and has very few paved roads, but is characterised primarily by extensive arable land interspersed with a dense network of canals, drains, ditches, rows of trees, and windbreaks. The entire territory is cultivated intensively, and the main crops are wheat, barley, tomatoes, and corn. It is the area with the lowest human population density in Italy.

The Valle del Mezzano is an important area for waterfowl and for wintering and nesting birds of prey. It also hosts a significant population of pheasant (*Phasianus colchicus*); additionally, many species of mammals are present, including the European hare (*Lepus europaeus*), the fox (*Vulpes vulpes*), the roe deer (*Capreolus capreolus*), the wolf (*Canis lupus*), and the coypu (*Myocastor coypus*). The most recent data on fox density date back to 2015, with densities of 0.37/kmq in the South-East area and 0.65/kmq in the North-West area (Regione Emilia–Romagna, unpublished data); No actual data are available for the abundance of pheasants and raptors. In the 1980s, the area supported a population of 16,000 partridges, which originated from the annual release of several hundred bred individuals that did not belong to the subspecies *Perdix p*. *italica*. This population has experienced a significant decline over the years, as in the rest of Italy and much of Europe, leading to local extinction in the early 2000s.

This SPA mainly features clayey soils rich in peat deposits and has a consistently shallow, mostly brackish groundwater to the east, supporting a distinctly halophilic spontaneous flora once crops are abandoned. The northern boundary of the site was recently incorporated into the Delta Po Regional Park. In the study area and within a radius of about 10 km, *Perdix perdix* spp. are absent, and the introduction of non-Italian grey partridge is banned.

Arable land accounted for 93% of the study area and was dominated by maize (20–25%), soybean (15%), wheat and barley (30–40%), ryegrass (5–10%), sugar beet (5%), and horticultural crops, including tomato, onion, melon, watermelon, and potato (20–25%). Crop proportions varied year on year. Inland wetlands accounted for 0.3%, water bodies for 2.1%, rivers and canals for 1.4%, and naturalised areas for 3.2% of the study area. The cultivated area plot size is about 150–180 ha, and the natural plot area is about 35–40 ha. Edges run along canal sides, with a width of about 30 m and a length of several kilometres. Data on field chemical treatments were not available; however, the entire Valle del Mezzzano is a Special Protection Area established under the UE Birds Directive for the protection of birds, and the use of field insecticides is regulated by the Regione Emilia–Romagna. The areas where release pens were built were not chemically treated.

Founder birds were selected based on mitochondrial DNA genetic features previously retrieved in museum samples collected in the wild in Italy before 1920, prior to the release of allochthonous individuals for game purposes [4]. To determine whether these historical features (haplotypes) persist in current wild and captive-bred populations, ISPRA’s Area for Conservation Genetics (BIO-CGE) gathered information and characterised the genetic composition of grey partridges from some remnant Italian wild populations and from several Italian breeding farms. Combining data from the genetic characterisation of specimens from both historical and current populations allowed us to describe the distribution of the haplotypes in Italy before and after release events. After identifying an appropriate stock of grey partridges in an Italian breeding facility, birds were translocated to the Bieri Wildlife Centre, managed by Carabinieri Forestali (CUFA). BIO-CGE provided the breeding farm with specific collection protocols, sampling kits, and on-site practical training for farm staff.

In 2019, more than 4000 samples were collected from the breeding farm, and their metadata were entered into the Perdix database. Mitochondrial DNA haplotypes were analysed in 2905 individuals, forming the breeding stock for the first year. This selection enabled the pairing of more than 250 reproductive couples. For the second year’s breeding stock, 699 individuals were analysed for mtDNA. The results of this screening allowed us to pair 274 reproductive couples. In 2021, breeding pairs were selected based on previous genetic selection and phenotypic characteristics.

### 2.3. Reintroduction

In the Valle del Mezzano, reintroduction was carried out using soft-release methods with release pens; this was an obligatory choice because of the large number of birds involved and the size of the project area (about 17,000 ha). The eighteen 18 release pens, each 0.5–0.6 ha, were built with the lower part of the fence electrified to exclude terrestrial predators. Five release aviaries (3.90 × 3.90 m) (for a total of 90 release aviaries), 10 feeders and 10 drinkers (5 internal to the release pens and 5 external) were installed in each enclosure, for a total of 180 feeders and 180 drinkers, which were refilled almost daily during the reintroduction period. Each release pen was equipped with a water cistern to supply the drinkers.

In each release pen, crops suitable for partridges were sown, with plant species selected according to the seasonal sowing period. Over the course of these 4 years, crops were sown in both spring and autumn. Sowing was carried out in alternating strips of alfalfa, millet, sorghum, sunflower, and other plant species preferred by partridges, to enhance their habitat and provide additional food sources. In this way, about 70–80% of the internal surface of any release pen was covered. Despite the particularly dry conditions in 2021, which hindered the growth of some crops, the presence of spontaneous herbs helped maintain a good level of vegetation cover. This natural growth partially compensated for the lack of coverage and provided suitable conditions for the partridges.

The reintroduction involved a total of 30,078 partridges, each individually identified with a leg ring, with suitable individuals supplied from the Bieri Wildlife Centre: 5438 in 2021, 10,040 in 2022, 9000 in 2023 and 5600 in 2024.

The reintroduction and release operations for *Perdix perdix italica* have been carried out using the soft-release method in each year (2021, 2022, 2023) from August to early October [13].

Partridges were introduced in an average of 3 rounds per release pen. Specifically, there were rounds of about 500, 750, and 1050 partridges. On average, about 30 animals were placed first in aviaries (acclimatisation cages) and, after 5–10 days, were released from the pens.

To improve the fitness of the founder Italian grey partridges, we exposed the young birds to aerial predators, buzzards (*Buteo buteo*), and terrestrial predators, red foxes (*Vulpes vulpes*), before reintroducing them to the Valle del Mezzano [15]. Furthermore, predator control of generalist predators (red foxes, coypus, magpies *Pica pica*, and carrion crow *Corvus cornix*) was implemented in the study area to reduce predation risk [16].

### 2.4. Radiotelemetry

A total of 241 animals were tagged over three years; both males and females were fitted with a VHF tag, model PIP3-Ag393, LOTEK Ltd. Wareham United Kingdom, 4.5 g (about 1.24% of the body mass of the radio-tracked birds) with an approximate battery life of 9 months. The VHF tags operated in the 149,000–151,000 MHz band. To prevent signal overlap among animals, distinct transmission frequencies were assigned to each transmitter, with a spacing of at least 10 kHz. The radiotelemetry study on reintroduced animals aimed to assess post-release survival, the distance travelled from the release pen, and to estimate home ranges after release. In 2021, 8 release pens were available, and 40 animals were tagged and tracked. In 2022 and 2023, 18 pens were available, and 130 and 71 animals were tagged and tracked, respectively. All tagged birds were kept in aviaries for 2 days by the operators to monitor their condition before being gradually released into the release pens.

To monitor the animals after release, we used the homing method, preferred over triangulation because of the study area’s wide-open, obstacle-free spaces, and to collect detailed data on survival, habitat use, and the presence of other grey partridges, including marked ones. However, homing techniques could alter the movement and behaviour of the tracked animals [17]; in our study, to prevent bias, we adopted a non-intensive sampling scheme.

During the first month of data collection, each animal was tracked three times a week. From the second month after release onwards, tracking was conducted once a week until the radio collar’s battery was depleted or the animal died. After locating the tagged grey partridge, we recorded the radio-collar frequency, the animal’s ID, date, time, weather conditions, homing position, reaction, geographical coordinates, group composition, habitat, and the operator who collected the data.

The fundamental assumption in statistical analyses of home range sizes is that locations are statistically independent [17,18]. If an animal is at point *x* at time *t*, the probability that it will be at a different point at time *t + k* increases as the interval *k* grows. Therefore, to obtain a random sample, locations must be spaced sufficiently in time so that each is uncorrelated with previous and subsequent locations. Autocorrelated data can distort estimates of an animal’s home range size, often yielding estimates lower than the actual size. Discontinuous sampling was thus performed, and to ensure independence, a time gap of more than 12 h was maintained between consecutive fixes for each animal. However, the typical sampling plan—taking one fix per animal three times a week—generally kept the interval between fixes well above the 12 h threshold.

The final dataset was divided by year of release, with all fixes collected through homing for each animal released that year. Preliminary data quality analysis and coordinate transformation were carried out in QGIS-LTR version 3.40.5, Bratislava [19].

The survival analysis was initially explored using the Kaplan–Meier method [20], as this method provides unbiased estimates in the presence of censored observations, as in the case of a staggered release programme [17].

Survival analyses were subsequently conducted using Cox proportional hazards models implemented in the survival package in R [21]. Sex and body mass at release were initially included as explanatory variables. Release year was included either as a fixed effect or as a stratification factor when proportional hazards assumptions were violated, and release site was included as a frailty term to account for site-level heterogeneity. Individuals alive at the end of the monitoring period or lost to monitoring were treated as right-censored observations. The proportional hazards assumption was assessed using Schoenfeld residuals (cox.zph). When proportionality assumptions were violated, time-dependent covariates or stratified Cox models were applied.

Post-release dispersal distance and home range metrics were analysed using generalized linear mixed models (GLMMs) with Gamma error distribution and log link implemented in the lme4 package. Sex, standardized body mass at release, and release year were included as fixed effects, while release site was included as a random intercept to account for site-level heterogeneity. Statistical analysis was performed using the “Lme4” R package [22].

The animals considered for home-range analysis were those with at least 20 fixes available during the data-collection period. Fixes from the first month were reduced from 3 to 1 per week for home-range analysis. The selection of which fix to consider for each week in the first month was made at random. Once the animals for which at least 20 fixes were available had been identified, three sub-datasets were created for subsequent analysis in R [23] using the ‘adehabitatHR 0.4.22’ package [24]. The home ranges of the reintroduced individuals were calculated using two methods: the minimum convex polygon (MCP) using 100% of locations, so as not to lose information about areas frequented by individuals, and the kernel density estimation (KDE) based on the concept of individual animals’ utilization distribution (UD). In this study, the KDE method was used to determine the 95% and 50% utilisation rates (defined as the core area) for individual animals. Given the relatively low number of locations available for several individuals, the reference bandwidth (href) implemented in the adehabitatHR 0.4.22 approach was considered more conservative and reduced the risk of underestimating space use.

The results from the MCP and KDE calculations were then imported into QGIS as shapefiles, and the areas occupied by individual animals were calculated in hectares (ha) using the UTM 32N zone.

Because MCP95 and KDE95 estimates were strongly influenced by sampling effort, these metrics were used only for descriptive comparisons with previous studies. Core area size was analysed using a generalised linear mixed model with a Gamma error distribution and a log link, including release year as a fixed effect and release site as a random intercept. Model assumptions for GLMMs were evaluated using simulated residual diagnostics in the DHARMa package, including inspection of residual distributions and dispersal tests. Continuous predictors were standardised when necessary to improve numerical stability and model convergence. Model complexity was reduced where required to avoid overparameterisation relative to sample size. Final models were selected based on biological relevance, model stability, convergence diagnostics, and the interpretability of parameter estimates.

### 2.5. Partridge Count

During the pre-reproductive period (March and April), grey partridge calling males were counted using the playback method. This approach is well established for enhancing the detectability of bird species with territorial calls, especially when spontaneous calling activity is low or inconsistent [23]. Since the species is monogamous, each male’s territory contains only one female, and the sex ratio can be assumed to be 1:1 [3]. In spring, males often establish and defend territories by singing, while females are less likely to respond [25]. Playback surveys effectively trigger territorial responses, increasing the likelihood of detecting individuals that might otherwise remain silent or hidden. They are considered a powerful tool in conservation studies [26]. In our survey, we recorded the coordinates and time of all the calling points, the call typology, weather conditions, the number of loudspeakers used and the researcher involved. However, the playback could alter the behaviour of birds, or they could vary their behavior in the presence of a predator; indeed, this method could produce a bias in the estimates of the population size [27]. For this reason we considered the results as a Density Index, not an absolute density.

**Figure 2:**
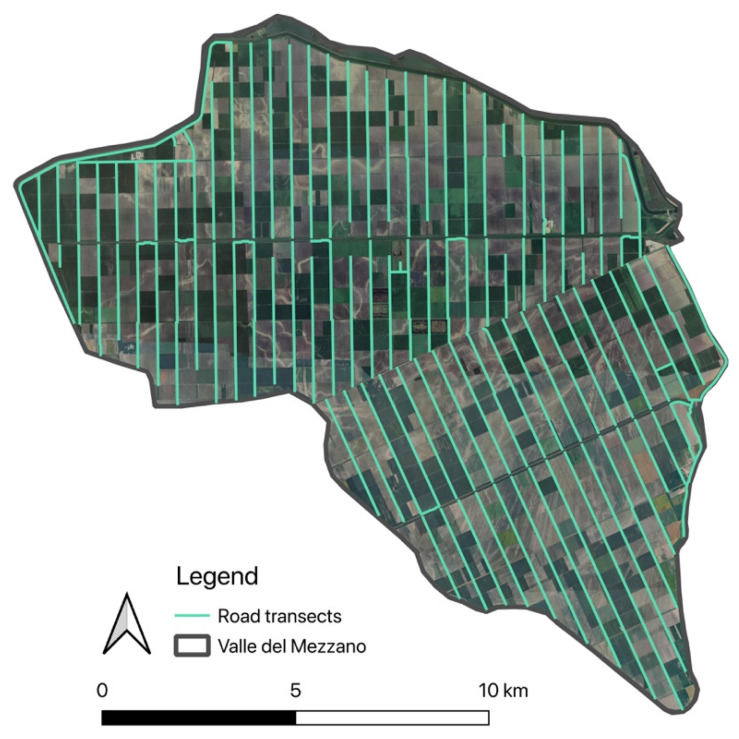
Road transects used in Valle del Mezzano for the spring playback survey of Italian grey partridge males.

The monitoring strategy relied on counting singing males of *Perdix perdix italica* to estimate both the minimum number of territorial males and their density in the surveyed area. Playback surveys were conducted at fixed listening points, georeferenced with GPS and spaced approximately 250 m apart along standardised routes (transects) (Figure 2). Each listening point was visited once per session.

For practical reasons and to cover the entire reintroduction area, these transects followed the main roads, drainage ditches, and secondary channels in the Valle del Mezzano. The census involved several operators from different project partners, each equipped with a portable loudspeaker system that played male territorial calls. Each session lasted approximately two weeks, depending on the number of operators and environmental and weather conditions. In April, monitoring was essential to confirm the number of birds and pairs contacted during the March session and to locate grey partridges during the breeding season. In 2022, monitoring was conducted at 1813 listening points (transect length 352.24 km) during the March session and at 1830 (350.95 km) during the April session. In 2023, the total number of listening points for both the April (358.43 km) and May (354.71 km) sessions was 1839. In 2024, the number of listening points was 1933 (331.37 km) in the March session and 1697 (331.76 km) in the April session. Variations in the total numbers across sessions were due to adverse weather or obstacles along the route.

The survey was conducted from dawn until around 11:00 a.m., except on a few days when it was interrupted by strong winds or other adverse weather. These interruptions, in fact, reduced the range of the recorded call, which is usually about 250 m [17]. The transects were covered by car, with stops at fixed listening points. Once the engine was turned off, the operator played the territorial call in all four directions inside the car with the windows down. Each call was followed by roughly 30 s of silence, during which the operator listened for any territorial response calls from the males.

Observers moved quietly and discreetly to avoid influencing bird behaviour. During listening periods, the surveyor carefully observed any grey partridge with binoculars, including those that might not have responded to the call but had been attracted by it.

For each contacted animal, data on the number of individuals, sex, and age classes (if identifiable), as well as the type of observation (male seen, male heard, pairs, and responses to playback calls), were recorded on field sheets, along with additional information on weather conditions, habitat, and time (start and end of the transect and each contact). The locations of the counted animals were documented on a map and via GPS, with UTM coordinates recorded on the field sheet.

## 3. Results

### 3.1. Survival and Dispersal

Of the 241 radiotracked partridges, 58 were excluded from dispersal and home range analysis due to collar failure or because they disappeared immediately after release and were not subsequently localised; however, the disappeared or lost birds without confirmation of death were treated as right-censored observations. In total, we analysed the survival of 241 and dispersal of 187 reintroduced birds (Table 1).

**Figure 3:**
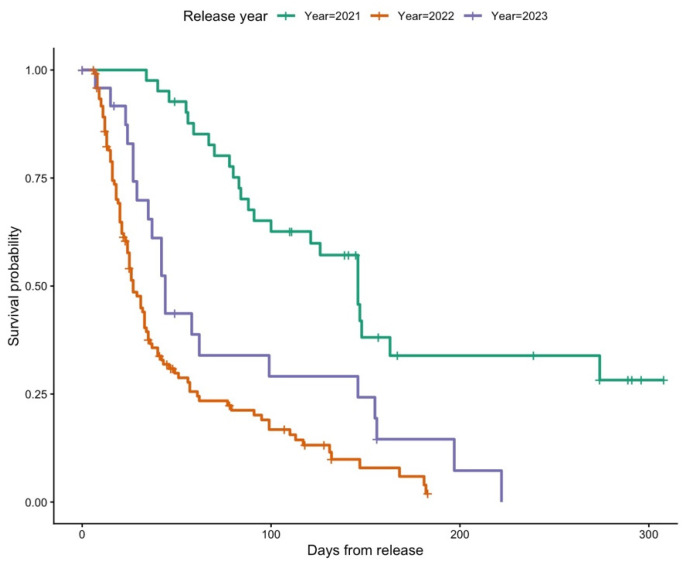
Survival of Italian grey partridges reintroduced to Valle del Mezzano; the Kaplan–Meier graph highlights, for each year of release, the decline in survival as a function of time since release.

A total of 143 out of 241 released birds (59.33%) were confirmed dead, with an overall survival of 40.67%; Figure 3 shows the survival curve for each release year. Survival estimates may be overestimated if some individuals classified as censored had in fact died shortly after signal loss. However, because signal loss may also result from transmitter failure or permanent emigration outside the monitored area, these cases could not be reliably classified as mortality events.

Predation was the major cause of mortality (n = 105): 85 (80.95%) were preyed on by raptors, 12 (11.43%) were preyed on by foxes and other mammalian predators, and the remaining 26 individuals by unknown predators.

Accounting for time-dependent effects and site-level heterogeneity, sex (HR = 1.13, 95% CI = 0.80–1.58, *p* = 0.47) did not significantly influence survival. The effect of body mass varied over time, but no consistent temporal pattern emerged (HR = 0.97, 95% CI = 0.97–1.03, *p* = 0.28).

**Figure 4:**
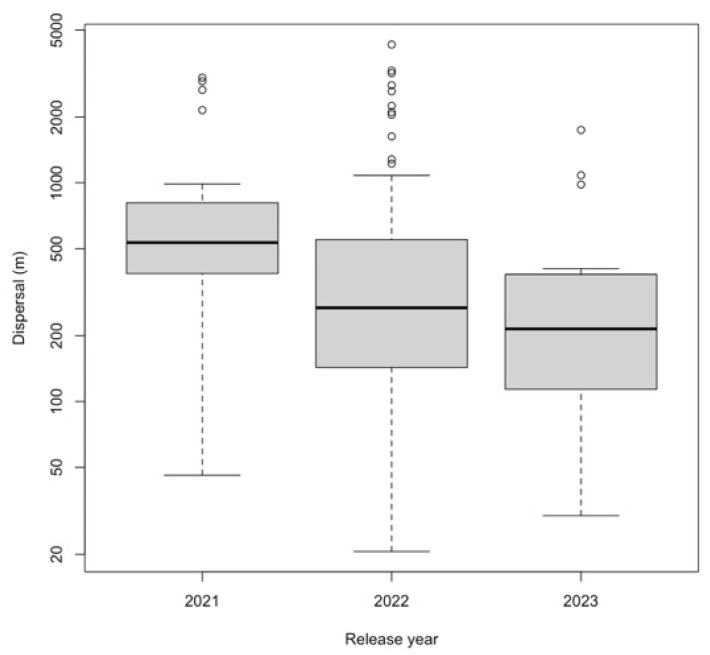
Box-plot chart for dispersal of Italian grey partridges reintroduced in different years to Valle del Mezzano.

The average dispersal distance of the 180 birds was 545.22 m (SD 690.25 m) with a maximum value of 4289.29 m; 68.33% of birds remained within a radius of 500 m from the release site, and only 7.22% dispersed more than 1.5 Km. Dispersal varied significantly across release years, whereas individual traits had weak or no effect (Table 2). Males tended to disperse farther than females, though the effect was weak and not statistically significant (β = 0.23, *p* = 0.09). Body mass at release did not influence dispersal distance (*p* = 0.69). However, birds released in 2023 dispersed significantly shorter distances than those released in the reference year (β = −0.80, *p* = 0.005) (Figure 4). Release enclosures contributed to variability in dispersal distance, supporting the inclusion of enclosures as a random effect. Finally, the model showed good convergence and accounted for site-level heterogeneity (random-intercept variance = 0.14).

### 3.2. Home Ranges

For all 21 Italian grey partridges with a location number of ≥20, we estimated the total home range (MCP 95%, KDE 95% and KDE 50%) (Table 3).

**Figure 5:**
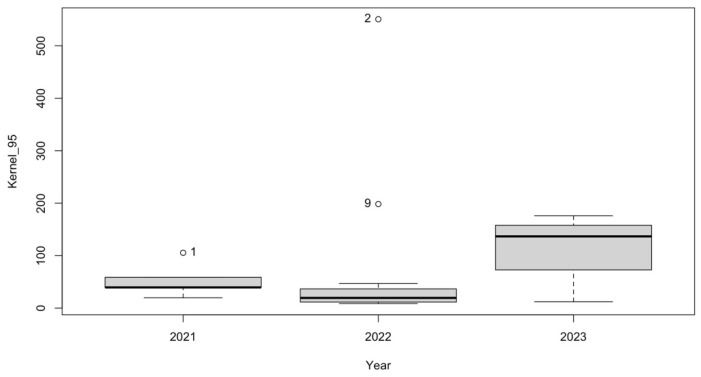
Box-plot chart for home range (KDE 95%) of Italian grey partridges released in different years in the Valle del Mezzano.

Home range size (MCP) of radio-tagged birds is highly variable, with values ranging from 3.74 to 113.88 ha and a mean value of 29.43 ha. Data indicate that mean MCP95 and KDE95 home range sizes differed across release years, with generally smaller home ranges observed in 2022 than in other years (Figure 5). However, MCP95 and KDE95 estimates were used descriptively and primarily to facilitate comparisons with previous studies.

Core area size was analysed using a generalised linear mixed model with a Gamma error distribution and a log link, including release year as a fixed effect and release site as a random intercept (Table 4).

Core home-range size differed across release years. Individuals released in 2022 exhibited significantly smaller core areas than in the reference year, whereas no clear difference was observed between the size of core areas observed in 2021 and 2023. Body mass did not influence core area size. The release site accounted for a substantial proportion of the variance in core area size.

### 3.3. Spring Counts

The results of spring playback monitoring are reported in Table 5.

The average number of males counted between 2022 and 2024 was 196.67 in the March session and 204.33 in the April session. However, in April, more couples were detected than in March, as pair formation is nearly complete by mid-spring in Italy.

Data showed a positive trend for both the observed couple and the overall number of males over the project years.

### 3.4. Density Estimate of Reproductive Males

Over the three years, we calculated a density index (DI), defined as the number of males observed per unit area (total transect length with a 250 m buffer on each side). The results (Table 6) show year-to-year variation, with the density index highest in 2024, the final year of the project, and lowest in 2023.

However, our index accounts only for animals that were actually contacted, representing a percentage of the suitable area for the partridge at Valle del Mezzano; furthermore, it should be considered that it is very improbable we detected all individuals within a 250 m radius.

Considering the average value of the yearly March and April sessions, the growth rate of DI is negative between the 2nd and 1st year (λ_23/22_ = 0.81) and positive between the 3rd and 4th year (λ_24/23_ = 1.73); in 2021, there were no wild reproductive pairs.

## 4. Discussion

The main finding of our study is the extremely high mortality among the radio-marked birds (59.33%), with predation the leading cause. Considering the possible bias due to using disappeared birds as right-censored in survival analysis, our observations align with those from other grey partridge reintroduction projects that have used artificially reared individuals (Table 7).

The most effective predators were birds of prey, as also reported by Parish and Sotherton at the Kirriemuir site [29] and highlighted by Carroll in other areas [3]; however, the red fox had a greater impact on grey partridges [32]. Our result could be explained by the high number of raptors breeding and wintering in the Valle del Mezzano, as well as by red fox control activity in this area. In accordance with the EU Birds Directive 2009/147/EC and the conservation status of the birds of prey, no measures to limit the numbers of nesting or wintering raptors were implemented.

As suggested by other authors, the high mortality of released captive-reared birds may be linked to their low fitness, poor predator-avoidance behavior, and maladaptive use of habitat structure [29,33].

The average dispersal values at the release site are close to those reported by Buner [31], with almost all individuals located within 1.5 km of the site. However, the dispersal values are lower than those reported by Putaala and Hissa [28] on the breeding and dispersal of natural and reintroduced grey partridges.

Home range sizes estimated using MCP95 and KDE95 were within the ranges reported for grey partridges in previous studies (Table 8), although direct comparisons should be made with caution, given differences in sampling effort and estimation methods.

Survival rates did not consistently vary by sex or body mass, whereas dispersal distances and core home range sizes differed by release year. Although release cohort size (numbers of released birds varied greatly in different years) may influence post-release spatial behaviour through density-dependent processes, model comparison suggested that annual variation in core area size was better explained by year-level effects than by release cohort size alone.

Notably, birds released in 2022 exhibited smaller core areas, implying more focused space use, possibly due to suitable habitat conditions or resource availability. Additionally, year-to-year differences in dispersal suggest that environmental conditions affected movement patterns after release. The significant variability across sites in dispersal and core ranges underscores the role of local habitat features in shaping spatial behaviour.

Although only a limited set of individual traits was evaluated, year and site-level variables consistently explained a larger proportion of variation in survival and spatial behaviour than the measured individual predictors. In fact, the reintroduced stock was very uniform: all birds were 90 days old and had been exposed to terrestrial and aerial predators before release. Additional behavioural, physiological, or genetic factors may nevertheless contribute to post-release performance and should be investigated in future studies.

Spring counts of singing males, considered as potential reproductive pairs, suggest the presence of reproductive individuals just after the first-year release. In fact, despite the high mortality observed in individuals equipped with radio collars, a consistent number of territorial singing males and pairs were contacted.

The 2024 density index (3.22 pairs/100 ha) of grey partridges in Valle del Mezzano is similar to the pair densities observed in the same area in 1984 and 1985, which were 5.63 and 3.10 pairs/100 ha, respectively. Similar values (min 2.3–6.7 pairs/100 ha) were also observed in a reintroduced population in central Italy [44].

Across the rest of Europe, modern-day population status is highly variable. In France, population abundance was highly variable across habitat types and management, with values ranging from <5 pairs/100 ha in marginal areas to >25 pairs/100 ha in the core population [45]. A long-term study in Poland highlights spring density ranging from 0.4 to 8.3 pairs/100 ha, after declining from 20.0 pairs/100 ha in 1991 [46]. In southern England, the spring populations are quite different between specifically managed areas for grey partridges (average of 13.2 pairs/100 ha) and the conventionally managed areas (1.3 pairs/100 ha) [16]. Low spring population density was also observed in Bulgaria over a 15-year period, with an average of 1.5 ± 1.83 pairs/100 ha [47]. In Serbia, the grey partridge faced a population decline [48]; however, spring density in a suburban habitat near Skopje ranged from 7.65 to 11.78 pairs/100 ha [49].

Our results reveal a population with low-to-medium spring densities, similar to those observed in other European study areas and well below the historical peaks of the last century. However, the population in Valle del Mezzano cannot be considered self-sustaining because releases continue.

Nevertheless, as opportunistic observations confirm, the species is breeding in Valle del Mezzano; In fact, direct observations made using pointing dogs in July and August 2023 and 2024 indicate reproductive success of a mean of 2.25 (n. observations = 40) and 3.25 (n. = 40) juveniles per breeding female [50].

Given the observed combination of very large release numbers, high predation pressure, and the ineffectiveness of mitigation strategies, there is a concrete possibility of a demographic sink maintained by continuous releases.

However, our findings have important implications for reintroduction management and conservation strategies to increase the success of Italian grey partridges.

As highlighted by Raintanen et al. [33,51], captive-bred grey partridges are often characterised by maladaptive behaviours (i.e., low vigilance against predators, feeding throughout the day rather than only at dawn and dusk, and selecting habitats unsuitable for predator avoidance). However, an improvement to the reintroduction method will be the fostering of young partridges by wild pairs [31]; this activity will be feasible only when the release of individuals ends, and there are individuals born in nature in the study area available.

Habitat structure surrounding release sites may play a crucial role in reducing excessive post-release movements and promoting local settlement. The availability of refuge cover, habitat heterogeneity, and suitable foraging areas within short distances from release points could reduce dispersal and potentially increase survival during the critical post-release phase. Accordingly, management actions aimed at improving vegetation cover and maintaining heterogeneous agroecosystems around release areas may enhance grey partridge reintroduction success.

Furthermore, habitat management is a key factor in the success of grey partridge conservation projects [13,52,53,54], and a significant portion of the intervention area, approximately 25–30%, should be managed to provide cover from predators and guarantee food for chicks and adults. This includes banning the use of field insecticides, protecting field margins, and planting beetle banks.

In particular, the most important factors for reducing predation on nesting grey partridges are providing sufficient nesting habitat size (>20 m in width) and promoting a high proportion of edge structures and high habitat diversity [55]. The linear edge length could reduce the predation risk because these structures distract terrestrial predators (red fox, Mustelids) from the core nesting habitat. Furthermore, grassland seems to offer greater protection against nesting predation due to more microhabitat selection options. On the other hand, grassland could become an ecological trap if they will be mowed during the grey partridge nesting and hatching period. Predation risk could also be reduced by placing a flower block (artificially created patches of annual and perennial flowering plants sown to increase habitat diversity) in a highly diversified habitat [56]. Flower plots managed without field pesticides, or using selective ones, also support a higher insect count and can enhance chick survival [13]. However, achieving this land-based approach requires the active participation of farmers and land managers [16,57].

Finally, wooded areas near the nesting grey partridge site increase predator presence because these habitats offer ample opportunities for raptors and corvids to nest and for mammals to build dens or resting sites [58]. Indeed, reintroduction projects should be carried out in areas with poor woodland or forest cover.

Other conservation actions could involve predator control, red foxes and carrion crows [29], as well as wild boar (*Sus scrofa*), which prey on eggs and chicks, thereby affecting the reproductive success of ground-nesting birds [59]. Furthermore, interspecific competition for nesting or feeding sites could have a negative impact on conservation; in fact, in sympatric areas, the red-legged partridge (*Alectoris rufa*) is competitively dominant over the grey partridge, shifting the latter away from its optimal ecological niche [60].

The upcoming Italian grey partridge conservation initiatives should be implemented in protected areas of at least 5000 hectares and should involve genetically selected birds. The conservation of Perdix perdix italica encounters more complex challenges because non-native individuals released into the wild for hunting are present. Therefore, the main approach is to regulate the introduction of grey partridges to reduce the risk of genetic pollution to the endemic genetic forms.

## 5. Conclusions

Like other reintroduction projects involving prey species and founders bred in captivity, our project faces very high post-release mortality, mainly due to raptor predation [20,23]. Furthermore, survival, dispersal, spatial behaviour, and spring density are more affected by stochastic factors than by sex or the release site of the founders. In particular, spring weather conditions could greatly influence grey partridges’ reproduction [35].

Despite the challenges faced, the Italian grey partridge has bred in the Valle del Mezzano after its extinction in the wild. However, to ensure the stabilization (without releases) of the population, it is necessary to continue monitoring and implement actions to enhance the species’ survival, such as controlling opportunistic predators and managing habitats specific to the grey partridge. Finally, based on our pilot project, other reintroduction projects of the Italian grey partridge in suitable protected areas should be carried out to ensure a large-scale meta-population approach [61].

At the national level, to conserve the grey partridge, it is essential to approve an action plan for the species, establish sustainable harvesting practices based on ongoing population monitoring, and implement targeted environmental management for the grey partridge.

## Figures and Tables

**Table 1:** Average survival (d) and dispersal distances (m) of male and female Italian grey partridge reintroduced to Valle del Mezzano during 2021–2023. Body mass is summarized for all birds radiotracked.

		Survival (Days)			Dispersal (m)			Body Mass (g)		
Year	Sex	n	Average	SD	n	Average	SD	n	Average	SD
2021	F	20	142.0	77.16	20	833.6	853.84	20	403.00	26.92
	M	21	131.4	78.30	21	643.2	506.17	21	412.75	40.15
2022	F	69	39.4	42.27	66	423.2	580.34	69	342.37	32.51
	M	61	39.3	41.25	56	629.2	846.71	61	355.00	32.04
2023	F	34	29.1	56.78	12	280.0	249.81	34	352.92	38.17
	M	31	20.1	43.34	12	418.7	529.63	31	355.00	26.55
TOTAL		241	52.3	66.02	187	545.2	690.25	241	362.19	41.01

**Table 2:** Dispersal of Italian grey partridges reintroduced to Valle del Mezzano, the results of the generalised linear mixed model fit by maximum likelihood: AIC = 2624.7, BIC = 2647.1, logLik −1305.4 df.resid = 173.

Random Effect			Variance	Std. Dev.
Release Pen			0.1438	0.3792
Residual			1.1682	1.0808
Fixed effect		Std Error	t value	*p*
Intercept	6.431	0.216	29.732	<0.001
Sex_M	0.233	0.139	1.679	0.09
Body mass	0.045	0.092	0.487	0.626
2022	0.378	0.235	−1.608	0.108
2023	0.791	0.283	−2.797	0.005

**Table 3:** Estimated total home ranges of the 21 tracked Italian grey partridges reintroduced to Valle del Mezzano (values in ha). 2021: 5 birds; 2022: 12 birds; 2023: 4 birds.

Year	Bird	Sex	Body Mass	N° Fix	MCP 95%	Kernel 95%	Kernel 50%
2021	ST006F	F	435	20	47.09	105.51	15.81
2021	ST040F	F	415	26	33.12	39.36	6.28
2021	ST008F	F	420	35	35.38	58.78	10.91
2021	ST012M	M	360	32	11.05	19.69	2.78
2021	ST007M	M	455	34	27.36	39.48	5.22
2022	ST080F	F	365	20	5.34	26.5	4.64
2022	ST087F	F	350	20	9.54	19.56	2.69
2022	ST096F	F	325	22	12.73	19.46	2.88
2022	ST148F	F	420	22	113.88	198.37	26.77
2022	ST086F	F	335	24	5.63	16.48	2.77
2022	ST069F	F	350	27	7.12	12.07	1.69
2022	ST062F	F	380	29	4.25	8.88	1.73
2022	ST157F	F	375	30	11.76	23.73	5.71
2022	ST151F	F	425	34	3.74	10.97	1.92
2022	ST058M	M	350	20	102.46	550.54	144.02
2022	ST072M	M	300	21	16.32	46.93	5.96
2022	ST128M	M	305	35	4.27	9.15	1.26
2023	ST208F	F	340	21	59.89	176.01	41.52
2023	ST231F	F	380	21	40.02	139.85	22.63
2023	ST200M	M	310	25	61.78	133.61	28.58
2023	ST206M	M	365	25	5.34	12.09	3.09

**Table 4:** Home range (KDE50) of the Italian grey partridge in Valle del Mezzano, the results of the generalised linear mixed model fit by maximum likelihood for core area: AIC = 163.3, BIC = 142.6, logLik 62.1 df.resid = 15.

Random Effect			Variance	Std. Dev.
Release Pen			1.532	1.2379
Residual			0.270	0.52
Fixed effect		Std Error	t value	*p*
Intercept	2.3	0.626	3.671	<0.001
Body mass	−0.164	0.273	−0.601	0.548
2022	−1.044	0.424	−2.462	0.013
2023	0.53	0.58	0.914	0.361

**Table 5:** Results of the Italian grey partridge spring counts in Valle del Mezzano (AKI: Abundance Kilometre Index).

Year	Period	Listening Stations	Singing Males	Observed Males	Observed Couples	Sex Not Determined	Total Males	AKI
2022	7–18 March	1830	66	26	75	8	171	0.49
	2–16 April	1813	63	16	101	4	182	0.52
2023	6–17 March	1839	54	9	85	8	152	0.42
	12–26 April	1839	40	19	112	3	173	0.49
2024	4–16 March	1933	89	20	147	21	267	0.81
	8–29 April	1697	61	30	165	3	258	0.78

**Table 6:** Density index of reproductive Italian grey partridge males during the spring counts in the Valle del Mezzano.

Year	Period	DI (Males/100 ha)
2022	7–18 March	1.94
	2–16 April Average	2.07 2.01
2023	6–17 March	1.70
	12–26 April Average	1.95 1.83
2024	4–16 March	3.22
	8–29 April Average	3.11 3.17

**Table 7:** Survival of reintroduced grey partridge in the Valle del Mezzano and other studies. ^1.^ Estimate of survival probability from the beginning of April to the median date of the onset of incubation; ^2.^ monthly survival calculated with a mark-recapture model; ^3.^ birds lost without confirmation of mortality were treated as right-censored observations.

Year	Area	Type	Survival (%)	Citation
1998	Finland ^1^	Wild hen in spring	68.6	[28]
		Released hen in spring	18.5	
2007	Great Britain	Autumn release	10	[29]
		Females spring release	30	
2008	Switzerland ^2^	Wild birds born in wild	90	[30]
		Fostered chick	86	
		Wild birds translocated	70	
2011	Switzerland	Young fostered	20	[31]
		Young unfostered	7	
		Adults in autumn	10	
		Adults in spring	9	
2021–2024	Italy ^3^	Released 90 days old	40.67	Present study

**Table 8:** Grey partridge home ranges in Valle del Mezzano and in previous studies.

Period	Area	Home Range (ha)	Birds	Citation
Adults without young in summer	North Dakota	71.9 ± 64.2	4	[3]
Autumn	South Dakota	16–310	4	[34]
Winter	North Dakota	116.6 ± 101.6	17	[35]
Winter	South Dakota	96 ± 110.6	8	[34]
Severe winter	North Dakota	4.9–34.0	8	[36]
Spring	Pyrenees	118	24	[37]
Summer	Pyrenees	126	19	[37]
Winter	Montana	1.4 (0.1–5.6)	-	[38]
Winter groups	Pianura Padana	7.92 ± 3.5	-	[39]
Breeding pairs	Pianura Padana	2.1 ± 1.3	-	[39]
Family groups	Pianura Padana	0.5 ± 2.6	-	[39]
Spring	Mezzano	25.5 ± 5.7	6	[40]
Family groups (chicks < 2 weeks)	North Dakota	8.2 ± 9.0	5	[41]
Family groups (chicks 2–4 weeks)	North Dakota	23.1 ± 17.7	3	[41]
Family groups with pre-fledge youngs	Wisconsin	19.7 ± 17.7	3	Cited in [3]
Pairs breeding season	Northeast England	3–64	35	[42]
Spring pairs	Southwest England	7.42 ± 1.60	29	[33]
Autumn coveys	Southwest England	25.43 ± 7.31	19	[33]
Winter ^1^	New York State	105 ± 7.6	7	[43]
Winter ^2^	New York State	157 ± 5.1–392 ± 41.0	9–15	[43]
Spring ^1^	New York State	82 ± 2.3	5	[43]
Spring ^2^	New York State	672 ± 37.5–304 ± 17.9	10–10	[43]
Autumn	Mezzano	29.43 ± 31.29	21	Present study

^1^ wild or established population, ^2^ reintroduction project.

## Data Availability

The database of GPS locations of tracked perdix and spring localisations of males is available from ISPRA.
